# A Global Workspace perspective on mental disorders

**DOI:** 10.1186/1742-4682-2-49

**Published:** 2005-12-21

**Authors:** Rodrick Wallace

**Affiliations:** 1 Epidemiology of Mental Disorders Research Dept., The New York State Psychiatric Institute, Box 47, 1051 Riverside Dr., New York, NY, 10032, USA

## Abstract

**Background:**

Recent developments in Global Workspace theory suggest that human consciousness can suffer interpenetrating dysfunctions of mutual and reciprocal interaction with embedding environments which will have early onset and often insidious staged developmental progression, possibly according to a cancer model, in which a set of long-evolved control strategies progressively fails.

**Methods and results:**

A rate distortion argument implies that, if an external information source carries a damaging 'message', then sufficient exposure to it, particularly during critical developmental periods, is sure to write a sufficiently accurate image of it on mind and body in a punctuated manner so as to initiate or promote similarly progressively punctuated developmental disorder, in essence either a staged failure affecting large-scale brain connectivity, which is the sine qua non of human consciousness, or else damaging the ability of embedding goal contexts to contain conscious dynamics.

**Conclusion:**

The key intervention, at the population level, is clearly to limit exposure to factors triggering developmental disorders, a question of proper environmental sanitation, in a large sense, primarily a matter of social justice which has long been known to be determined almost entirely by the interactions of cultural trajectory, group power relations, and economic structure, with public policy. Intervention at the individual level appears limited to triggering or extending periods of remission, representing reestablishment of an extensive, but largely unexplored, spectrum of evolved control strategies, in contrast with the far better-understood case of cancer.

## Introduction

Mental disorders in humans are not well understood. Indeed, such classifications as the *Diagnostic and Statistical Manual of Mental Disorders – fourth edition*, [1], the standard descriptive nosology in the US, have been characterized as 'prescientific' by Gilbert [2] and others. Arguments from genetic determinism fail, in part because of an apparently draconian population bottleneck which, early in our species' history, resulted in an overall genetic diversity less than that observed within and between contemporary chimpanzee subgroups. Arguments from psychosocial stress fare better, but are affected by the apparently complex and contingent developmental paths determining the onset of schizophrenia – one of the most prevalent serious mental disorders – dementias, psychoses, and so forth, some of which may be triggered in utero by exposure to infection, low birthweight, or other stressors. Our own work suggests that many sleep disorders may also be broadly developmental [3].

Gilbert suggests an extended evolutionary perspective, in which evolved mechanisms like the 'flight-or-fight' response are inappropriately excited or suppressed, resulting in such conditions as anxiety or post traumatic stress disorders. Nesse [4] suggests that depression may represent the dysfunction of an evolutionary adaptation which down-regulates foraging activity in the face of unattainable goals.

Kleinman and Good, however, ([5], p. 492) have outlined some of the cross cultural subtleties affecting the study of depression which seem to argue against any simple evolutionary interpretation:

"When culture is treated as a constant (as is common when studies are conducted in our own society), it is relatively easy to view depression as a biological disorder, triggered by social stressors in the presence of ineffective support, and reflected in a set of symptoms or complaints that map back onto the biological substrate of the disorder... However, when culture is treated as a significant variable, for example, when the researcher seriously confronts the world of meaning and experience of members of non-Western societies, many of our assumptions about the nature of emotions and illness are cast in sharp relief. Dramatic differences are found across cultures in the social organization, personal experience, and consequences of such emotions as sadness, grief, and anger, of behaviors such as withdrawal or aggression, and of psychological characteristics such as passivity and helplessness or the resort to altered states of consciousness. They are organized differently as psychological realities, communicated in a wide range of idioms, related to quite varied local contexts of power relations, and are interpreted, evaluated, and responded to as fundamentally different meaningful realities... Depressive illness and dysphoria are thus not only interpreted differently in non-Western societies and across cultures; they are *constituted *as fundamentally different forms of social reality."

More generally, Kleinman and Cohen [6] find that

" [S]everal myths... have become central to psychiatry... The first is that the forms of mental illness everywhere display similar degrees of prevalence... [Second is] an excessive adherence to a principle known as the pathogenic/pathoplastic dichotomy, which holds that biology is responsible for the underlying structure of a malaise, whereas cultural beliefs shape the specific ways in which a person experiences it. The third myth maintains that various unusual culture-specific disorders whose biological bases are uncertain occur only in exotic places outside the West... In an effort to base psychiatry in 'hard' science and thus raise its status to that of other medical disciplines, psychiatrists have narrowly focused on the biological underpinnings of mental disorders while discounting the importance of such 'soft' variables as culture and socioeconomic status..."

Further, serious mental disorders in humans are often comorbid among themselves – depression and anxiety, compulsive behaviors, psychotic ideation, etc. – and with serious chronic physical conditions such as coronary heart disease, atherosclerosis, diabetes, hypertension, dyslipidemia, and so on. These too are increasingly recognized as developmental in nature (see [7,8] for references), and are frequently compounded by behavioral problems like violence or substance use and abuse. Indeed, smoking, alcohol and drug addiction, compulsive eating, and the like, are often done as self-medication for the impacts of psychosocial and other stressors, constituting socially-induced 'risk behaviors' which synergistically accelerate a broad spectrum of mental and physical problems.

Recent research on schizophrenia, dyslexia, and autism, supports a 'brain connectivity' model for these disorders which is of considerable interest from a global workspace perspective, since large-scale brain connectivity is essential for the operation of consciousness, a principal, and very old, evolutionary adaptation in higher animals.

Burns et al. [9], on the basis of sophisticated diffusion tensor magnetic resonance imaging studies, find that schizophrenia is a disorder of large-scale neurocognitive networks rather than specific regions, and that pathological changes in the disorder should be sought at the supra-regional level. Both structural and functional abnormalities in frontoparietal networks have been described and may constitute a basis for the wide range of cognitive functions impaired in the disorder, such as selective attention, language processing and attribution of agency.

Silani et al. [10] find that, for dyslexia, altered activation observed within the reading system is associated with altered density of grey and white matter of specific brain regions, such as the left middle and inferior temporal gyri and left arcuate fasciculus. This supports the view that dyslexia is associated with both local grey matter dysfunction and with altered [larger scale] connectivity among phonological/reading areas.

Villalobos et al. [11] explore the hypothesis that large-scale abnormalities of the dorsal stream and possibly the mirror neuron system, may be responsible for impairments of joint attention, imitation, and secondarily for language delays in autism. Their empirical study showed that those with autism had significantly reduced connectivity with bilateral inferior frontal area 44, which is compatible with the hypothesis of mirror neuron defects in autism. More generally, their results suggest that dorsal stream connectivity in autism may not be fully functional.

Courchesne and Pierce [12] suggest that, for autism, connectivity within the frontal lobe is excessive, disorganized, and inadequately selective, whereas connectivity between frontal cortex and other systems is poorly synchronized, weakly responsive and information impoverished. Increased local but reduced long-distance cortical-cortical reciprocal activity and coupling would impair the fundamental frontal function of integrating information from widespread and diverse systems and providing complex context-rich feedback, guidance and control to lower-level systems.

Coplan [13] has observed a striking pattern of excessive frontal lobe self-connectivity in certain cases of anxiety disorder, and Coplan et al. [14] find that maternal stress can affect long-term hippocampal neurodevelopment in a primate model.

As stated, brain connectivity is the sine qua non of the Global Workspace model of consciousness, and further analysis suggests that these disorders cannot be fully understood in the absence of a functional theory of consciousness, and in particular, of a detailed understanding of the elaborate regulatory mechanisms which must have evolved over the past half billion years to ensure the stability of that most central and most powerful of adaptations.

Distortion of consciousness is not simply an epiphenomenon of the emotional dysregulation which many see as the 'real' cause of mental disorder. Like the pervasive effects of culture, distortion of consciousness lies at the heart of both the individual experience of mental disorder and the effect of it on the embedding of the individual within both social relationships and cultural or environmental milieu. Distortion of consciousness in mental disorders inhibits both routine social interaction and the ability to meet internalized or expected cultural norms, a potentially destabilizing positive feedback. Distortion of consciousness profoundly affects the ability to learn new, or change old, skills in the face of changing patterns of threat or opportunity, perhaps the most critical purpose of the adaptation itself. Distortion of consciousness, particularly any decoupling from social and cultural context, is usually a threat to long-term individual survival, and those with mental disorders significantly affecting consciousness typically face shortened lifespans.

This paper will review some recent advances in consciousness theory, and apply the results toward a refocus on the role of that adaptation in mental disorders, using an information theory formalism which draws a parallel between punctuated evolutionary and cognitive/learning forms of information transmission [15]. The method stands in contrast to neural network studies of mental disorder, (e.g. [16]). As Krebs [17] has argued, neural network models of mental function fall victim to a 'sufficiency indeterminacy' in the same sense that the Ptolemaic system of astronomy, with its endless epicycle-upon-epicycle Fourier series expansion of planetary dynamics, fails in comparison with the Newtonian analysis of central gravitational motion. That is, as Krebs puts it, neural possibility does not imply neural plausibility, and neural network computer models of mental phenomena can be constructed to do literally whatever one wants, in the same sense that a Fourier series can be constructed to approximate any function over a fixed interval without providing much basic understanding of that function.

Our comparison of punctuated evolutionary adaptation with cognitive learning plateaus is counterintuitive: evolution is not a cognitive process. Cognition involves an active selection of one out of a complex repertory of possible responses to a sensory input, based on comparison with a learned representation of the outer world (e.g. [18,19]). Although genes, or in the case of human biology, a composite of genes-and-culture (e.g. [20]), do indeed constitute a kind of 'memory' of past interaction with the world, response to selection pressure is not through direct comparison with that 'memory', but rather through the reproductive success of a random variation constrained by the path of evolutionary history. This is not cognition, and there can be no 'intelligent purpose' to adaptive or evolutionary process. Nonetheless, selection pressures represent systematic patterns of interaction with an embedding and highly structured ecosystem in which each species is itself manifest. We will, below, use this perspective to infer a rough analog between developmental onset and progression of a broad class of mental disorders and the onset and progression of a certain class of cancers.

Recent resumption of scientific research on consciousness in humans follows from Baars' [21] pioneering restatement of the problem in terms of a global workspace theory [21,22], to which the reader should refer for more details.

The central ideas are as follows [22]:

(1) The brain can be viewed as a collection of distributed specialized networks (processors).

(2) Consciousness is associated with a global workspace in the brain – a fleeting memory capacity whose focal contents are widely distributed (broadcast) to many unconscious specialized networks.

(3) Conversely, a global workspace can also serve to integrate many competing and cooperating input networks.

(4) Some unconscious networks, called contexts, shape conscious contents, for example unconscious parietal maps modulate visual feature cells that underlie the perception of color in the ventral stream.

(5) Such contexts work together jointly to constrain conscious events.

(6) Motives and emotions can be viewed as goal contexts.

(7) Executive functions work as hierarchies of goal contexts.

Although this basic approach has been systematically elaborated upon for nearly twenty years by a number of quite eminent researchers, consciousness studies has only recently, in the context of a deluge of data from brain imaging experiments, come to the point of actually digesting the perspective and moving on.

The Baars model has received increasing experimental verification over the last two decades (e.g. [23,24]). Since it particularly attempts to properly represent the matter of embedding and interpenetrating contexts, it provides a basis for understanding the synergism of consciousness and mental disorders in humans, in particular the role of embedding social and cultural contexts, and for drawing a parallel with the initiation and progression of cancer as a disorder of information, which is more fully discussed in [25].

My own recent work provides a rigorous mathematical formulation of the GW blackboard model, in terms of an iterated, second-order, contextually-embedded, hierarchical General Cognitive Model (GCM) crudely analogous to hierarchical regression. It is, however, based on the Shannon-McMillan rather than on the Central Limit Theorem, and is strongly supplemented by methodologies from topological manifold theory and differential geometry [3,8,26]. Recent results [26] suggest that, in fact, it should be possible to make a rigorous theory of 'all possible' GW blackboard models, much in the same sense that the Church lambda calculus describes 'conventional' computers and the Nix/Vose Markov chain treatment describes many possible genetic algorithms [27,28].

We begin with a simplified analysis focusing on modular networks of interacting cognitive substructures, and particularly study the importance of their embedding in progressively larger systems. More complicated examples, involving renormalization treatment of phase transitions affecting information sources, iterated to second order, can be found in [8].

## The simplest modular network global workspace model

### Cognition as 'language'

Cognition is not consciousness. Indeed, most mental, and many physiological, functions, while cognitive in a particular formal sense, hardly ever become entrained into the Global Workspace of consciousness. For example, one seldom is able to consciously regulate immune function, blood pressure, or the details of binocular tracking and bipedal motion, except to decide 'what shall I look at', 'where shall I walk'. Nonetheless, many cognitive processes, conscious or unconscious, appear intimately related to 'language', broadly speaking. The construction is surprisingly straightforward [8,29].

We begin the formal analysis with a very general, and hence deceptively 'weak', mathematical treatment of cognitive process [8,29].

Atlan and Cohen [19] and Cohen [18] argue, in the context of immune cognition, that the essence of cognitive function involves comparison of a perceived signal with an internal, learned picture of the world, and then, upon that comparison, choice of one response from a much larger repertoire of possible responses.

Cognitive pattern recognition-and-response, from this view, proceeds by functionally combining an incoming 'external sensory signal' with an internal 'ongoing activity', incorporating the learned picture of the world, and triggering some appropriate action based on a decision that the pattern of sensory activity requires a response. An explicit neural network example is given in Wallace ([8] pp. 34–36).

More formally, a pattern of sensory input is mixed in an unspecified but systematic manner with a pattern of internal ongoing activity to create a path of combined signals *x *= (*a*_0_, *a*_1_, ..., *a*_*n*_, ...). Each *a*_*k *_thus represents some algorithmic composition of internal and external signals.

This path is fed into a highly nonlinear, but otherwise similarly unspecified, nonlinear decision oscillator which generates an output *h*(*x*) that is an element of one of two disjoint sets *B*_0 _and *B*_1 _of possible system responses. Let

*B*_0 _≡ *b*_0_, ..., *b*_*k*_,

*B*_1 _≡ *b*_*k*+1_, ..., *b*_*m*_.

Assume a graded response, supposing that if

*h*(*x*) ∈ *B*_0_,

the pattern is not recognized, and if

*h*(*x*) ∈ *B*_1_,

the pattern is recognized, and some action *b_j_, k *+ 1 ≤ *j ≤ m *takes place.

Again, for concrete examples see [8], pp. 34–36.

The principal objects of interest are paths *x *which trigger pattern recognition-and-response exactly once. That is, given a fixed initial state *a*_0_, such that *h*(*a*_0_) ∈ *B*_0_, we examine all possible subsequent paths *x *beginning with *a*_0 _and leading exactly once to the event *h*(*x*) ∈ *B*_1_. Thus *h*(*a*_0_, ..., *a*_*j*_) ∈ *B*_0 _for all *j < m*, but *h*(*a*_0_, ..., *a*_*m*_) ∈ *B*_1_. Wallace [8] examines the possibility of more complicated schemes as well.

For each positive integer *n*, let *N*(*n*) be the number of high probability 'grammatical' and 'syntactical' paths of length *n *which begin with some particular *a*_0 _having *h*(*a*_0_) ∈ *B*_0 _and lead to the condition *h*(*x*) ∈ *B*_1_. Call such high probability paths 'meaningful', assuming, not unreasonably, that *N*(*n*) will be considerably less than the number of all possible paths of length *n *leading from *a*_0 _to the condition *h*(*x*) ∈ *B*_1_. To reiterate, details of, and more elaborate justifications for, this approach are to be found in [8].

While combining algorithm, the form of the nonlinear oscillator, and the details of grammar and syntax, can all remain unspecified in this model, the critical mathematical assumption which permits inference on necessary conditions is that the finite limit



both exists and is independent of the path *x*.

We call such a pattern recognition-and-response cognitive process *ergodic*. Not all cognitive processes are likely to be ergodic, implying that *H*, if it indeed exists at all, is path dependent, although extension to 'nearly' ergodic processes is possible [8].

Invoking the spirit of the Shannon-McMillan Theorem, it is possible to define an adiabatically, piecewise stationary, ergodic information source **X **associated with stochastic variates *X_j _*having joint and conditional probabilities *P*(*a*_0_, ..., *a*_*n*_) and *P *(*a*_*n*_|*a*_0_, ..., *a*_*n*-1_) such that appropriate joint and conditional Shannon uncertainties satisfy the classic relations



This information source is defined as *dual *to the underlying ergodic cognitive process [8].

The Shannon uncertainties *H*(...) are cross-sectional law-of-large-numbers sums of the form -Σ_*k *_*P*_*k *_log[*P*_*k*_], where the *P*_*k *_constitute a probability distribution. See [30-32] for the standard details.

### The giant component

A formal equivalence class algebra (and hence a groupoid, sensu Weinstein [33]) can be constructed by choosing different origin points *a*_0 _and defining equivalence by the existence of a high probability meaningful path connecting two points. Disjoint partition by equivalence class, analogous to orbit equivalence classes for dynamical systems, defines the vertices of the proposed network of cognitive dual languages. Each vertex then represents a different information source dual to a cognitive process.

We now suppose that linkages can fleetingly occur between the ordinarily disjoint cognitive modules defined by this algebra. In the spirit of [8], this is represented by establishment of a non-zero mutual information measure between them: cross-talk.

Wallace [8] describes this structure in terms of fixed magnitude disjunctive strong ties which give the equivalence class partitioning of modules, and nondisjunctive weak ties which link modules across the partition, and parametizes the overall structure by the average strength of the weak ties, to use Granovetter's [34] term. By contrast the approach here, initially, is to simply look at the average number of fixed-strength nondisjunctive links in a random topology. These are obviously the two analytically tractable limits of a much more complicated regime which we believe ultimately includes 'all possible' global workspace models.

Since we know nothing about how the cross-talk connections can occur, we will – for purposes of illustration only – assume they are random and construct a random graph in the classic Erdos/Renyi manner. Suppose there are *M *disjoint cognitive modules – *M *elements of the equivalence class algebra of languages dual to some cognitive process – which we now take to be the vertices of a possible graph.

As Corless et al. [35] discuss, when a graph with *M *vertices has *m *= (1/2)*aM *edges chosen at random, for *a > *1 it almost surely has a giant connected component having approximately *gM *vertices, with

*g*(*a*) = 1 + *W*(*-a *exp(*-a*))*/a*,     (2)

where *W *is the Lambert-W function defined implicitly by the relation

*W*(*x*) exp(*W*(*x*)) = *x. *    (3)

**Figure 1:**
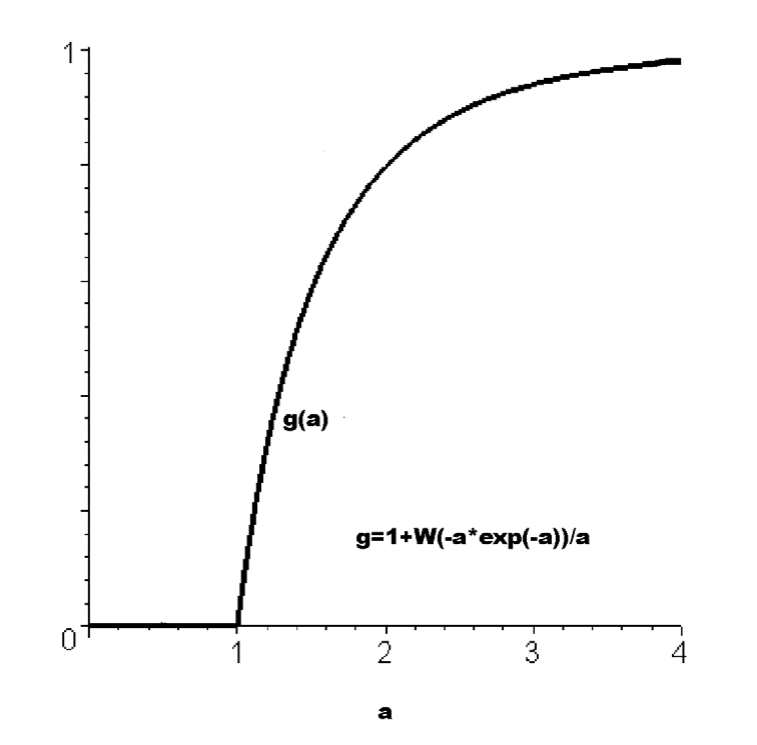
Relative size of the largest connected component of a random graph, as a function of 2× the average number of fixed-strength connections between vertices. *W *is the Lambert-W function, or the ProductLog in Mathematica, which solves the relation *W*(*x*) exp[*W*(*x*)] = *x*. Note the sharp threshold at *a *= 1, and the subsequent topping-out.'Tuning' the giant component by changing network topology generally leads to a family of similar curves, those having progressively higher threshold having correspondingly lower asymptotic limits (e.g. [41], fig. 4).

Figure 1 shows *g*(*a*), displaying what is clearly a sharp phase transition at *a *= 1.

Such a phase transition initiates a new, collective, shifting, cognitive phenomenon: the Global Workspace, a tunable blackboard defined by a set of cross-talk mutual information measures between interacting unconscious cognitive submodules. The source uncertainty, *H*, of the language dual to the collective cognitive process, which defines the richness of the cognitive language of the workspace, will grow as some function of *g*, as more and more unconscious processes are incorporated into it. Wallace [8] examines what, in effect, are the functional forms *H *∝ exp(*αg*), *α *ln [1/(1*-g*)], and (1/(1*-g*))^*δ*^, letting *R *= 1/1 *- g *define a 'characteristic length' in the renormalization scheme. While these all have explicit solutions for the renormalization calculation (mostly in terms of the Lambert-W function), other, less tractable, expressions are certainly plausible, for example *H *∝ *g^γ^,γ *> 0, *γ *real.

Given a particular *H*(*g*), the quite different approach of [8] involves adjusting universality class parameters of the phase transition, a matter requiring much mathematical development.

By contrast, in this new class of models, the degree of clustering of the graph of cognitive modules might, itself, be tunable, producing a variable threshold for consciousness: a topological shift, which should be observable from brain-imaging studies. Second order iteration would lead to an analog of the hierarchical cognitive model of [8].

Wallace [8] focuses on changing the average strength of weak ties between unconscious submodules rather than the average number of fixed-strength weak ties as is done here, and tunes the universality class exponents of the phase transition, which may also imply subtle shifts in underlying topology.

Following Albert and Barabasi ([36], Section V), we note that real networks differ from random graphs in that their degree distribution, the probability of *k *linkages between vertices, often follows a power law *P*(*k*) ≈ *k*^-*γ *^rather than the Poisson distribution of random networks,

*P*(*k*) = *a*^*k *^exp(*-a*)/*k*!, *k *≥ 0. Since power law networks do not have any characteristic scale, they consequently termed scale-free.

It is possible to extend the Erdos/Renyi threshold results to such 'semi-random' graphs. For example, Luczak [37] has shown that almost all random graphs with a fixed degree smaller than 2 have a unique giant cluster. Molloy and Reed [38,39] proved that, for a random graph with degree distribution *P*(*k*), an infinite cluster emerges almost surely when



Following Volz, [40], cluster tuning of random networks leads to a counterintuitive result. Define the clustering coefficient *C *as the proportion of triads in a network out of the total number of potential triads, i.e.



where *N*_Δ _is the number of triads in the network and *N*_3 _is the number of connected triples of nodes, noting that in every triad there are three connected nodes. Taking the approach of Molloy and Reed [38,39], Volz [40] shows quite directly that, for a random network with parameter *a*, at cluster value *C*, there is a critical value given by



If *C *= 0, i.e. no clustering, then the giant component forms when *a *= 1. Increasing *C raises *the average number of edges which must be present for a giant component to form. For *C *≥ , which is precisely the Golden Section, where the denominator in this expression vanishes, no giant component can form, regardless of *a*. Not all network topologies, then, can actually support a giant component, and hence, in this model, consciousness. This is of some importance, having obvious and deep implications ranging from the evolutionary history of consciousness to the nature of sleep.

A more complete exploration of the giant component can be found, e.g. in Newman et al. [41], especially the discussion leading to their figure 4. In general, 'tuning' of the GC will generate a family of curves similar to figure 1, but with those having threshold to the right of that in the plot 'topping out' at limits progressively less than 1: higher thresholds seem usually to imply smaller giant components. In sum, the giant component is itself highly tunable, replicating, in this model, the fundamental stream of consciousness.

Note that we do not, in this paper, address the essential matter of how the system of interacting cognitive modules behaves away from critical points, particularly in the presence of 'external gradients'. Answering this question requires the imposition of generalized Onsager relations, which introduce complications of topological 'rate distortion manifolds', metric structures, and the like (e.g. [3,8]).

### Mutual and reciprocal interaction: evading the mereological fallacy

Just as a higher order information source, associated with the GC of a random or semirandom graph, can be constructed out of the interlinking of unconscious cognitive modules by mutual information, so too external information sources, for example in humans the cognitive immune and other physiological systems, and embedding sociocultural structures, can be represented as slower-acting information sources whose influence on the GC can be felt in a collective mutual information measure. The measure will, through the Joint Asymptotic Equipartition Theorem which generalizes the Shannon-McMillan Theorem, be the splitting criterion for high and low probability joint paths across the entire system.

The tool for this is network information theory ([32], p. 387). Given three interacting information sources, *Y*_1_, *Y*_2_, *Z*, the splitting criterion, taking *Z *as the 'external context', is given by

*I*(*Y*_1_, *Y*_2_|*Z*) = *H*(*Z*) + *H*(*Y*_1_|*Z*) - *H*(*Y*_1_, *Y*_2_, *Z*),     (7)

where *H*(..|..) and *H*(.., .., ..) represent conditional and joint uncertainties [30-32].

This generalizes to



If we assume the Global Workspace/GC/blackboard to involve a very rapidly shifting, and indeed highly tunable, dual information source *X*, embedding contextual cognitive modules like the immune system will have a set of significantly slower-responding sources *Y*_*j*_, *j *= 1..*m*, and external social, cultural and other 'environmental' processes will be characterized by even more slowly-acting sources *Z*_*k*_, *k *= 1..*n*. Mathematical induction on equation (8) gives a complicated expression for a mutual information splitting criterion between high and low probability joint paths which we write as

*I*(*X*|*Y*_1_, .., *Y*_*m*_|*Z*_1_, .., *Z*_*n*_).     (9)

This encompasses a fully interpenetrating 'biopsychosociocultural' structure for individual consciousness, one in which Baars' contexts act as important, but flexible, boundary conditions, defining the underlying topology available to the far more rapidly shifting global workspace [3,8].

This result does not commit the mereological fallacy which Bennett and Hacker [42] impute to excessively neurocentric perspectives on consciousness in humans, that is, the mistake of imputing to a part of a system the characteristics which require functional entirety.

## Punctuation phenomena for information systems

As quite a number of researchers have noted, in one way or another, -see [8] for discussion – equation (1),



is homologous to the thermodynamic limit in the definition of the free energy density of a physical system. This has the form



where *F *is the free energy density, *K *the inverse temperature, *V *the system volume, and *Z*(*K*) is the partition function defined by the system Hamiltonian.

Wallace [8] shows at some length how this homology permits the natural transfer of renormalization methods from statistical mechanics to information theory. In the spirit of the Large Deviations Program of applied probability theory, this produces phase transitions and analogs to evolutionary punctuation in systems characterized by piecewise, adiabatically stationary, ergodic information sources. These 'biological' phase changes appear to be ubiquitous in natural systems and can be expected to dominate machine behaviors as well, particularly those which seek to emulate biological paradigms. Wallace [15] uses these arguments to explore the differences and similarities between evolutionary punctuation in genetic and learning plateaus in neural systems. Punctuated phenomena will emerge as important in the discussions below of subtle information system malfunctions, be those systems biological, social, or mechanical.

## The second order iteration

Suppose the giant component of the modular network associated with the Global Workspace of consciousness at some 'time' *k *is characterized by a set of parameters *A*_*k *_≡ , ..., . Fixed parameter values define a particular giant component structure. Suppose that, over a sequence of 'times' the giant component can be characterized by a path *x*_*n*_= *A*_0_, *A*_1_, ..., *A*_*n*-1 _having significant serial correlations which, in fact, permit definition of an adiabatically, piecewise stationary, ergodic (APSE) information source in the sense of [8]. Call that information source **X**. Suppose, again in the manner of [8], that a set of (external or else internal, systemic) signals impinging on consciousness, i.e. the giant component, is also highly structured and forms another APSE information source **Y **which interacts not only with the system of interest globally, but specifically with the tuning parameters of the giant component characterized by **X**. **Y **is necessarily associated with a set of paths *y*_*n*_.

Pair the two sets of paths into a joint path *z_n _*≡ (*x*_*n*_, *y*_*n*_), and invoke some inverse coupling parameter, *K*, between the information sources and their paths. By the arguments of the section above, this leads to phase transition punctuation of *I*[*K*], the mutual information between **X **and **Y**, under either the Joint Asymptotic Equipartition Theorem, or, given a distortion measure, under the Rate Distortion Theorem. *I*[*K*] is a splitting criterion between high and low probability pairs of paths, and partakes of the homology with free energy density described above. Attentional focusing then itself becomes a punctuated event in response to increasing linkage between the organism or device and an external structured signal, or some particular system of internal events. This iterated argument parallels the extension of the General Linear Model into the Hierarchical Linear Model of regression theory.

Call this the Hierarchical Cognitive Model (HCM).

## The dysfunctions of consciousness and intelligence: a cancer model

What is missing from this picture so far, and indeed will prove central, is the elaborate control mechanisms which must exist to ensure the integrity of the relation defined by equation (9), so that the information source *X*, representing the Global Neuronal Workspace of consciousness, remains confined to the topological structures defined by the external contexts represented by the set of information sources *Z*(*k*), *k *= 1...*n*. As a reviewer has noted, some mental disorders, at least, might be identified with the failure of these (social, cultural, and emotional) goal contexts to successfully constrain conscious events. Others may act by affecting the basic ability of the brain to engage in important large-scale coordinated activities.

More generally, equation (9), informed by the homology with equation (10), permits general discussion of the failure modes of global workspace systems of all kinds, in particular of their second order iteration which appears to be the analog to consciousness in higher animals.

The foundation for this lies in the Rate Distortion Theorem. Under the conditions of that theorem, equation (9) is the splitting criterion between high and low probability joint paths defining the maximum rate at which an external information source can write an image of itself having a given maximum of distortion, according to some defined measure [32,43]. Inverting the argument, equation (9) suggests that an external information source can, if given enough time, write an image of itself upon consciousness. That is, structures in *Z*-space can write images of themselves on *X*. If that external source is pathogenic, then, given sufficient exposure, some measure of consciousness dysfunction becomes inevitable.

This may not, in fact, be fully separate from the question of the pathological decoupling of *X *from the *Z*, as pathologies in *Z*-space may write an image of themselves onto the very containment mechanisms which are supposed to confine consciousness to the topology defined by cultural, social, and emotional goal contexts, ensuring the integrity of equation (9).

A more general discussion of comorbid mind/body disorders in humans emerges quite naturally [7]. The picture, in humans, then, is of a multifactorial and broadly interpenetrating mind/body/sociocultural dysfunction, often having early onset and insidious, irregular, developmental progression. These disorders are, broadly speaking, distorted images of pathogenic external environments which are literally written upon the developing embryo, on the growing child, and on the maturing adult ([8], Ch. 6). Equation (9) suggests that, in similar form, these images will be inevitably written upon consciousness as well, possibly through the failure of the mechanisms which are supposed to constrain consciousness to the embedding goal contexts.

Further consideration implies critical parallels with the initiation and progression of cancer in multicellular organisms, a quintessential disorder of information transmission.

The analogy requires some development, which is condensed from the information dynamics analysis of [25].

Nunney [44] suggests that in larger animals, whose lifespans are proportional to about the 4/10 power of their cell count, prevention of cancer in rapidly proliferating tissues becomes more diffcult in proportion to their size. Cancer control requires the development of additional mechanisms and systems with increasing cell count to address tumorigenesis as body size increases – a synergistic effect of cell number and organism longevity.

As Nunney puts it [44],

"This pattern may represent a real barrier to the evolution of large, long-lived animals and predicts that those that do evolve... have recruited additional controls [over those of smaller animals] to prevent cancer."

In particular different tissues may have evolved markedly different tumor control strategies. All of these, however, are likely to be energetically expensive, permeated with different complex signaling strategies, and subject to a multiplicity of reactions to signals.

Work by Thaler [45] and Tenaillion et al. [46] suggests that the mutagenic effects associated with a cell sensing its environment and history could be as exquisitely regulated as transcription. Invocation of the Rate Distortion or Joint Asymptotic Equipartition Theorems in address of the mutator necessarily means that mutational variation comes to significantly reflect the grammar, syntax, and higher order structures of embedding environmental processes. This involves far more than a simple 'colored noise' – stochastic excursions about a deterministic 'spine' – and most certainly implies the need for exquisite regulation. Thus there are deep information theory arguments in favor of Thaler's speculation.

Thaler [45] further argues that the immune system provides an example of a biological system which ignores conceptual boundaries between development and evolution.

Thaler specifically examines the meaning of the mutator for the biology of cancer, which, like the immune system it defies, is seen as involving both development and evolution.

Thus Thaler, in essence, looks at the effect of structured external stress on tumorigenesis and describes the 'local evolution' of cancer within a tissue in terms of a 'punctuated interpenetration' between a tumorigenic mutator mechanism and an embedding cognitive process of mutation control, including but transcending immune function.

The mutation control process constitutes the Darwinian selection pressure determining the fate of the (path dependent) output of a mutator mechanism. Externally-imposed and appropriately structured environmental signals then jointly increases mutation rate while decreasing mutation control effectiveness through an additional level of punctuated interpenetration. This is envisioned as a single, interlinked biological process.

Various authors have argued for 'non-reductionist' approaches to tumorigenesis (e.g. [47,48]), including psychosocial stressors as inherent to the process [49]. What is clear is that, once a mutation has occurred, multiple systems must fail for tumorigenesis to proceed. It is well known that processes of DNA repair (e.g.[50]), programmed cell death – apoptosis – (e.g. [51]), and immune surveillance (e.g. [52]) all act to redress cell mutation. The immune system is increasingly viewed as cognitive, and is known to be equipped with an array of possible remediations [18,19]. It is, then, possible to infer a larger, jointly-acting 'mutation control' process incorporating these and other cellular, systemic, and, in higher animals, social mechanisms. This clearly must involve comparison of developing cells with some internal model of what constitutes a 'normal' pattern, followed by a choice of response: none, repair, programmed cell death, or full-blown immune attack. The comparison with an internal picture of the world, with a subsequent choice from a response repertoire, is, as Atlan and Cohen [19] point out, the essence of cognition.

One is led to propose, in the sense of equation (9), that a mutual information may be defined characterizing the interaction of a structured system of external selection pressures with the 'language' of cellular cognition effecting mutation control. Under the Joint Asymptotic Equipartition or Rate Distortion Theorems, that mutual information constitutes a splitting criterion for pairwise linked paths which may itself be punctuated and subject to sudden phase transitions.

Pathologically structured externally environmental signals can become jointly and synergistically linked both with cell mutation and with the cognitive process which attempts to redress cell mutation, enhancing the former, degrading the latter, and significantly raising the probability of successful tumorigenesis.

Raised rates of cellular mutation which quite literally reflect environmental pressure through selection's distorted mirror do not fit a cognitive paradigm: The adaptive mutator may propose, but selection disposes. However, the effect of structured environmental stress on both the mutator and on mutation control, which itself constitutes the selection pressure facing a clone of mutated cells, connects the mechanisms. Subsequent multiple evolutionary 'learning plateaus' [15] representing the punctuated interpenetration between mutation control and clones of mutated cells constitute the stages of disease. Such stages arise in the context of an embedding system of environmental signals which, to use a Rate Distortion argument, literally writes an image of itself on all aspects of the disease.

These speculations are consistent with, but suggest extension of, a growing body of research. Kiecolt-Glaser et al. [53], for example, discuss how chronic inflammation related to chronic stress has been linked with a spectrum of conditions associated with aging, including cardiovascular disease, osteoporosis, arthritis, type II diabetes, certain cancers, and other conditions. Dalgleish [54,55] and others [56,57] have argued at length that chronic immune activation and inflammation are closely related to the etiology of cancer and other diseases. As Balkwill and Mantovanni [58] put the matter, "If genetic damage is the 'match that lights the fire' of cancer, some types of inflammation may provide 'fuel that feeds the flames' ".

Dalgleish [54] has suggested application of non-linear mathematics to examine the role of immune response in cancer etiology, viewing different phenotypic modes of the immune system – the Th1/Th2 dichotomy – as 'attractors' for chaotic processes related to tumorigenesis, and suggests therapeutic intervention to shift from Th2 to Th1. Such a shift in phenotype might well be viewed as a phase transition.

This analysis implies a complicated and subtle biology for cancer in higher animals, one in which external environmental 'messages' become convoluted with both pathogenic clone mutation and with an opposing, and possibly organ-specific, variety of tumor control strategies. In the face of such a biology, anti-inflammants [59] and other 'magic bullet' interventions appear inadequate, a circumstance having implications for control of the aging of conscious systems which we infer from these examples.

Although chronic inflammation, related certainly to structured environmental stress, is likely to be a contributor to the enhancement of pathological mutation and the degradation of corrective response, it is unlikely to be the only such trigger. The constant cross-talk between central nervous, hormonal, immune, and tumor control systems in higher animals guarantees that the 'message' of the external environment will write itself upon the full realm of individual physiology in a highly plieotropic, punctuated, manner, with multifactorial impact on both cell clone mutation and tumor control.

## Discussion and conclusion

These examples, particularly the model of cancer as an information disorder [25], suggest that consciousness in higher animals, the quintessence of information processing, is necessarily accompanied by elaborate regulatory and corrective processes, both internal and external, to ensure both the integrity of large-scale brain connectivity and that the dynamics of the Global Workspace are confined to the topology determined by embedding goal contexts. Only a few are well known: Sleep enables the consolidation and fixation in memory and semiautomatic mechanism of what has been consciously learned, and proper social interaction enhances mental fitness in humans. Other long-evolved, but currently poorly understood, mechanisms probably act as correctives to keep Gilbert's evolutionary structures from going off the rails, e.g. attempting to limit flight-or-fight HPA responses to 'real' threats, and so on.

Consciousness, a very old adaptation central to the survival of higher animals, has had the benefit of several hundred million years of evolution to develop the corrective and compensatory structures for its stability and efficiency over the life course. Although these are currently not well characterized, it seems clear that the synergism between culture and depression that Kleinman and others see as particularly characteristic of the disorder emerges 'naturally' from equation (9).

The explicit inference, then, is that human consciousness can suffer interpenetrating dysfunctions of mutual and reciprocal interaction with embedding environments which will have early onset and often insidious staged developmental progression, possibly according to a cancer model. These may affect general brain connectivity or act to degrade the linkage between consciousness and goal contexts, thus decoupling their topologies. There will be no simple, reductionist brain chemical 'bug in the program' whose 'fix' can fully correct such problems. On the contrary, the growth of an individual over the life course, and contact with toxic features of the outside world, can be expected to initiate developmental disorders which will become more intrusive over time, most obviously following some damage accumulation model, but likely according to far more subtle schemes, perhaps analogous to punctuated equilibrium in evolutionary context [15].

The obvious rate distortion argument suggests that, if an external information source is pathogenic, then sufficient exposure to it, especially during critical developmental periods, is sure to write a sufficiently accurate image of it on mind and body in a punctuated manner so as to initiate or promote similarly progressively punctuated developmental dysfunction. Critical periods include, but are surely not limited to, the uterine environment and early childhood. Sufficient trauma, particularly when exacerbated by post-traumatic secondary victimization, may be expected to trigger subsequent developmental dysfunction at any time.

To reiterate, these considerations suggest strongly that, in parallel with consciousness, an elaborate system of correction and control must have evolved as well, quite analogous to the elaborate tumor control mechanisms that necessarily accompany multicellularity.

As is the case with many cancers, the key health intervention, at the population level, is clearly to limit pathogenic exposures, a matter of proper environmental sanitation, in a broad sense, largely a question of social justice which has long been understood to be primarily determined by the interactions of cultural trajectory, group power relations, and economic structure with public policy (e.g. [60-65]).

At the individual level, intervention against mental disorders is likely to be far more problematic, just as it is in the treatment of cancer, and for analogous reasons. This analysis suggests that one does not often cure developmental disorders once they have begun, indeed, a good analogy seems to be with the onset of chronic organic disease. It is not at all easy to turn back the developmental clock, and the parallels with punctuated equilibrium in evolutionary process suggests a one-way street. The best that can often be achieved is to trigger or extend remission, either by encouraging or strengthening mechanisms of natural control and recovery, or through direct chemical or other intervention. That is, proper treatment can sometimes induce long-lasting remission, but this is subject to the determinants of medical failure, which are often precisely the factors of cultural trajectory, group power relations, economic structure, and public policy which are at the base of many population-level disease patterns. For an extended discussion see, for example, Wallace and Wallace [66], who find that structured psychosocial stress can write a literal image of itself onto the success or failure of individual-level therapeutic intervention, drug-related or not, just as it can on the development and functioning of human intelligence and consciousness.

Although one might argue, as with the immune system, that exposure to 'adverse environments' is necessary to develop resistance to induction of mental disorders, it seems likely that this depends on the degree of adversity and whether that adversity occurs at a developmental critical point in life history. Failure of maternal attachment in infancy due to external socioeconomic stressors has proved to have long-term consequences for hippocampal neurodevelopment in animal models [14].

We have begun to outline a consciousness-centered perspective on mental disorders, both those determined by defects in large-scale brain connectivity, and possibly by related failures of embedding goal contexts to constrain the topological dynamics of the global workspace. Further work in this direction might well focus on characterizing specific disorders from this viewpoint, and designing experiments to test such characterizations.

Equation (9) and the arguments surrounding it, however, already provide, in the context of mental disorders, quite a 'hard science' basis for the evolutionary anthropologist Robert Boyd's oft-repeated assertion that "culture is as much a part of human biology as the enamel on our teeth."

Not only culture and socioeconomic status, but historical trajectory, power relations between individuals and groups, and the effects of public policy, will write images of themselves onto the fundamental topology of individual consciousness, too frequently defining paths of debilitating developmental disorder which can blight lives.
